# Assessment of the effect of sphingosine kinase inhibitors on apoptosis,unfolded protein response and autophagy of T-cell acute lymphoblastic leukemia cells; indications for novel therapeutics

**DOI:** 10.18632/oncotarget.2318

**Published:** 2014-08-06

**Authors:** Cecilia Evangelisti, Camilla Evangelisti, Gabriella Teti, Francesca Chiarini, Mirella Falconi, Fraia Melchionda, Andrea Pession, Alice Bertaina, Franco Locatelli, James A. McCubrey, Dong Jae Beak, Robert Bittman, Susan Pyne, Nigel J. Pyne, Alberto M. Martelli

**Affiliations:** ^1^ Department of Biomedical and Neuromotor Sciences (DIBINEM), University of Bologna, Bologna, Italy; ^2^ Institute of Molecular Genetics, National Research Council-Rizzoli Orthopedic Institute, Bologna, Italy; ^3^ Muscoloskeletal Cell Biology Laboratory, IOR, Bologna, Italy; ^4^ Pediatric Oncology and Hematology Unit ‘Lalla Seragnoli’, S. Orsola-Malpighi Hospital, University of Bologna, Bologna, Italy; ^5^ Oncoematologia Pediatrica, IRCCS Ospedale Pediatrico Bambino Gesù, Rome, Italy; ^6^ Department of Microbiology and Immunology, Brody School of Medicine, East Carolina University, Greenville, NC, USA; ^7^ Department of Chemistry and Biochemistry, Queens College, The City University of New York, Flushing, New York, United States; ^8^ Strathclyde Institute of Pharmacy and Biomedical Sciences, University of Strathclyde, 161 Cathedral St, Glasgow, G4 0RE, Scotland, UK

**Keywords:** T-cell acute lymphoblastic leukemia, sphingosine kinase inhibitors, apoptosis, autophagy, unfolded protein response

## Abstract

Sphingosine 1-phosphate (S1P) is a bioactive lipid that is formed by the phosphorylation of sphingosine and catalysed by sphingosine kinase 1 (SK1) or sphingosine kinase 2 (SK2). Sphingosine kinases play a fundamental role in many signaling pathways associated with cancer, suggesting that proteins belonging to this signaling network represent potential therapeutic targets. Over the last years, many improvements have been made in the treatment of T-cell acute lymphoblastic leukemia (T-ALL); however, novel and less toxic therapies are still needed, especially for relapsing and chemo-resistant patients. Here, we analyzed the therapeutic potential of SKi and ROMe, a sphingosine kinase 1 and 2 inhibitor and SK2-selective inhibitor, respectively. While SKi induced apoptosis, ROMe initiated an autophagic cell death in our *in vitro* cell models. SKi treatment induced an increase in SK1 protein levels in Molt-4 cells, whereas it activated the endoplasmic reticulum (ER) stress/unfolded protein response (UPR) pathway in Jurkat and CEM-R cells as protective mechanisms in a sub-population of T-ALL cells. Interestingly, we observed a synergistic effect of SKi with the classical chemotherapeutic drug vincristine. In addition, we reported that SKi affected signaling cascades implicated in survival, proliferation and stress response of cells. These findings indicate that SK1 or SK2 represent potential targets for treating T-ALL.

## INTRODUCTION

The bioactive lipid sphingosine 1-phosphate (S1P) is implicated in many physiological and pathological processes such as survival, cell growth and migration [1]. On the contrary, its precursors, namely ceramide and sphingosine, are considered pro-apoptotic and anti-mitotic agents. As S1P, sphingosine and ceramide are interconvertible, it has been proposed that the balance of this “sphingolipid rheostat” is critical in determining cell death or survival [2]. S1P is formed by the phosphorylation of sphingosine by sphingosine kinase 1 (SK1) or sphingosine kinase 2 (SK2). SK1 has an important role in cancer [1], as demonstrated by the finding that its expression is upregulated at mRNA transcript and protein levels in numerous types of solid cancers, including lung cancers [3, 4] glioblastoma [5], thyroid cancer [6], and breast carcinoma [7, 8]

Many studies reported that high tumor SK1 expression is associated with increased disease progression, chemo-resistance and reduced patient survival [5, 7, 9, 10]. Furthermore, a role of SK1 has been recently proposed by Watson and colleagues in regulating the Warburg effect in prostate cancer cells [11].

On the other hand, the role of SK2 in regulating apoptosis is still a matter of debate, even though emerging evidence has highlighted the importance of a role for SK2 in cancer. Some studies supported a pro-apoptotic role of SK2 [12], while others demonstrated that selective SK2 inhibitors or siRNA silencing of SK2 induced cell death *in vitro* and *in vivo* [13, 14]. In addition, silencing of SK2 enhanced doxorubicin-induced apoptosis in breast or colon cancer cells [15]. Therefore, it appears evident that SKs represent a promising target for cancer therapy and increasing efforts are being made to develop isoform-selective inhibitors of SKs.

T-cell acute lymphoblastic leukemia (T-ALL) represents a malignant disorder arising from the neoplastic transformation of T-cell progenitors. T-ALL accounts for 10-15% of pediatric and 25% of adult cases [16]. The prognosis of pediatric T-ALL has recently improved due to intensified therapies, attaining more than 75% cure rates for children. However, pediatric T-ALL is prone to early relapse, and the prognosis of relapsed and primary chemo-resistant patients is poor [16]. Hence, more efficient and new therapeutic strategies displaying less toxicity are now required. Recently, the relevance of S1P in hematological malignancies has been highlighted by several groups [17, 18]. Importantly, a link between the S1P pathway and major signaling pathways aberrantly activated in T-ALL, such as phosphatidylinositol 3-kinase (PI3K)/Akt/mammalian target of rapamycin (mTOR) and Ras/Raf/MEK/ERK cascades has been described [19]. For these reasons, we decided to analyze the possible therapeutic effects of two SK inhibitors in T-ALL cell lines and primary cells: 2-(*p*-hydroxyanilino)-4-(*p*-chlorophenyl)thiazole (SKi), an SK1/2 inhibitor, and (*R*)-FTY720 methyl ether (ROMe), a SK2-selective inhibitor [20].

We reported herein that SKi and ROMe affected T-ALL cell viability; however, they exerted their effects through different mechanisms. Indeed, we showed here that the SK2 selective inhibitor ROMe induced an autophagic cell death, while SKi induced apoptosis. Moreover, for the first time, we demonstrated that SKi activated an ER stress/UPR pathway in a sub-population of T-ALL cells, a signaling network induced by the accumulation of unfolded proteins within the ER. In certain T-ALL cell lines, the UPR induced by SKi was linked with a protective autophagic response that appeared to be an attempt by the cells to counteract apoptosis induced by SKi. Finally, we demonstrated that SKi is synergistic with the conventional chemotherapeutic agent vincristine, suggesting that combined treatment of established chemotherapeutic agents with SK inhibitors could be a feasible approach to achieve efficacy in the treatment of T-ALL in the clinic. These results suggest that SK1 or SK2 inhibition is linked with a complex network of cell survival and death responses. Our findings provide information that may open new avenues for therapeutics designed to induce T-ALL cell death and thereby enable better management of this cancer.

## RESULTS

### SKi displays cytotoxic effects on T-ALL cell lines and patient T-ALL cells

**Figure 1:**
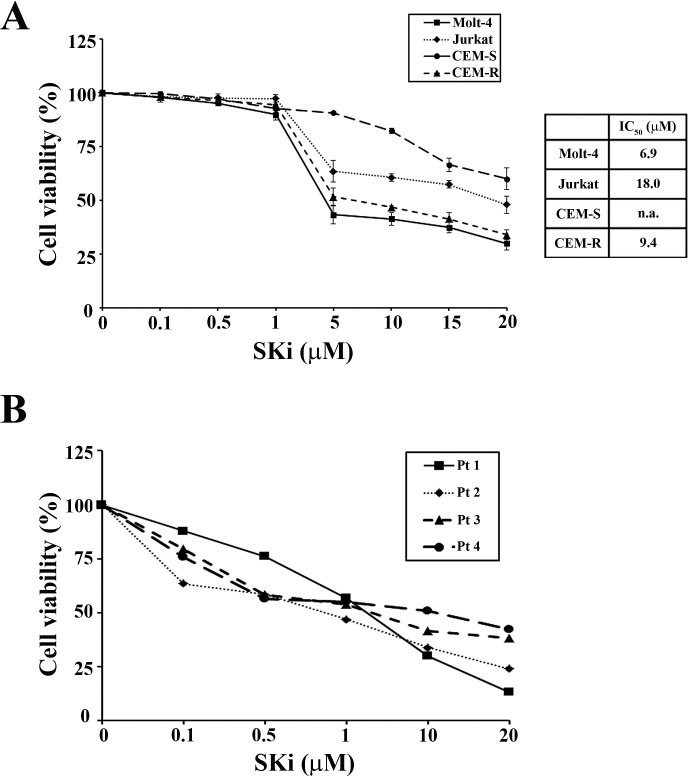
SKi affects viability of T-ALL cell lines and primary lymphoblasts (A) MTT assays were performed after 40 h of SKi treatment. Results are the mean of at least three different experiments ± s.d. Corresponding IC_50_ values are reported for each cell line in the table. The IC_50_ value for each data point was calculated with the appropriate software (CalcuSyn). (B) MTT assays on four representative patient samples treated for 24 h. n.a.: not attained.

Previous studies have shown that SKi displayed anti-proliferative and cytotoxic effects in acute and chronic myeloid leukemia cell lines [21]. To determine whether SKi also inhibited T-ALL cell proliferation and survival, we performed MTT assays using Molt-4, Jurkat, CEM-S and the drug-resistant CEM-R cell lines. Treatment of these cells with increasing concentrations of SKi for 40 h induced a reduction in cell viability with IC_50_ values of 6.9, 18, and 9.4 μM in Molt-4, Jurkat and CEM-R cells (Figure 1A). The IC_50_ for CEM-S cells was not attained within the SKi concentration range used in this study. SKi also affected cell viability of primary lymphoblasts from 4 different T-ALL pediatric patients with IC_50_ ranging from 0.79 to 5.5 μM (Figure 1B). In common with the T-ALL cell lines, a sub-population of three of the patient T-ALL cells appeared more resistant to SKi. Overall, these data demonstrated that SKi reduced the viability of T-ALL cell lines and primary human patient cells. This is likely due to the induction of cell death as we did not detect an impairment of the cell cycle at any time of cell treatment with SKi (data not shown).

### SKi promotes apoptosis in Molt-4 cell lines

**Figure 2:**
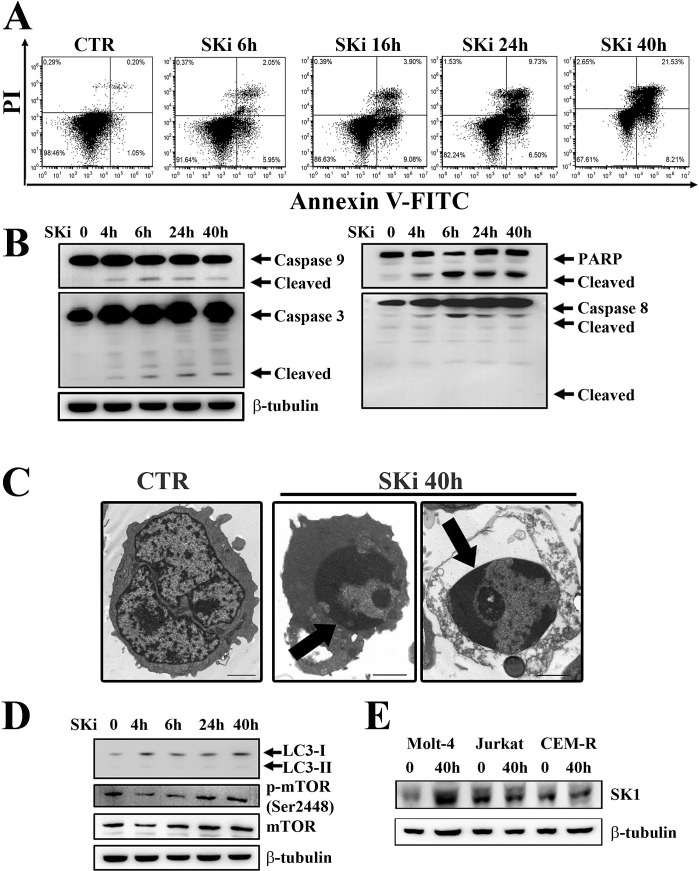
SKi triggers apoptosis in Molt-4 cell line (A) Flow cytometric analysis of Annexin-V-FITC/PI stained Molt-4 cells treated with SKi (6.9 μM) for 6, 16, 24 and 40 h documented a time-dependent increase in apoptotic cells with respect to untreated cells. (B) After SKi treatment, cells were collected, lysed and analyzed by western blotting for cleaved caspase-9, -3, and -8. (C) Molt-4 cells were treated with SKi (6.9 μM) for the indicated time, then processed for TEM analysis that documented apoptosis induction. Arrows point to condensed apoptotic chromatin. Scale bar 2 μm. (D) Western blot analysis for the autophagic marker LC3 and p-mTOR (Ser2448) in cells treated with SKi for the indicated times. (E) Western blot analysis for SK1 expression in T-ALL cell lines upon SKi treatment for 40 h. In (B) (D) and (E) 50 μg of protein was loaded for each lane. β-tubulin was used as a loading control.

In order to determine whether the decreased viability was related to apoptosis, Molt-4 cells were treated with a SKi concentration equivalent to the IC_50_ (6.9 μM) for 6, 16, 24 and 40 h, after which the cells were stained with Annexin-V and propidium iodide (PI) and analyzed by flow cytometry. Cells underwent apoptosis after 6 h of SKi treatment as demonstrated by the increasing presence of Annexin-V^+^/PI^−^ and Annexin-V^+^/PI^+^ cells which reflect early and late apoptosis, respectively (Figure 2A). The presence of low levels of PI-positive only cells after 40 h of treatment demonstrated that cell death was mainly due to apoptosis.

We next measured the activation status of various caspases. In this regard, activation of caspase -9, -3, and -8, as well as cleavage of poly ADP-ribose polymerase (PARP) was evident in SKi-treated cells in a time-dependent manner (Figure 2B). Transmission electron microscopy (TEM) analysis highlighted the presence of condensed apoptotic chromatin confirming apoptosis induction (Figure 2C). Furthermore, western blot analysis of LC3 protein, a widely recognized marker of autophagy, demonstrated that the autophagic process was not activated in Molt-4 cells (Figure 2D). This is consistent with the unchanged expression levels of Ser 2448 p-mTOR, a negative regulator of autophagy and whose decreased expression might be related to autophagy induction [22].

It has been reported that chronic exposure to SKi induced the proteasomal degradation of SK1 at protein level in solid tumor cancer cell lines [23]. Thus, we investigated whether a treatment of T-ALL cells with SKi might regulate the expression of SK1. Molt-4, Jurkat and CEM-R cells were treated with SKi for 40 h and SK1 expression was measured at both transcript and protein levels. SK1 transcript did not significantly change in all three cell lines (data not shown). SKi had no effect on SK1 protein levels in Jurkat and CEM-R cells; however, SKi induced an increase in SK1 protein levels in Molt-4 cells (Figure 2E). As high SK1 expression and S1P levels confer enhanced growth and survival to cells [5, 24, 25], the increase in SK1 protein levels could represent an attempt of Molt-4 cells to escape SKi-induced apoptosis. Indeed, a sub-population of Molt-4 cells do appear more resistant to high concentrations (> 5 μM) of SKi (Figure 1A), where there is no further decline in cell viability.

### SKi induced an ER-stress-dependent autophagy to protect cells against apoptosis in Jurkat and CEM-R cell lines

**Figure 3:**
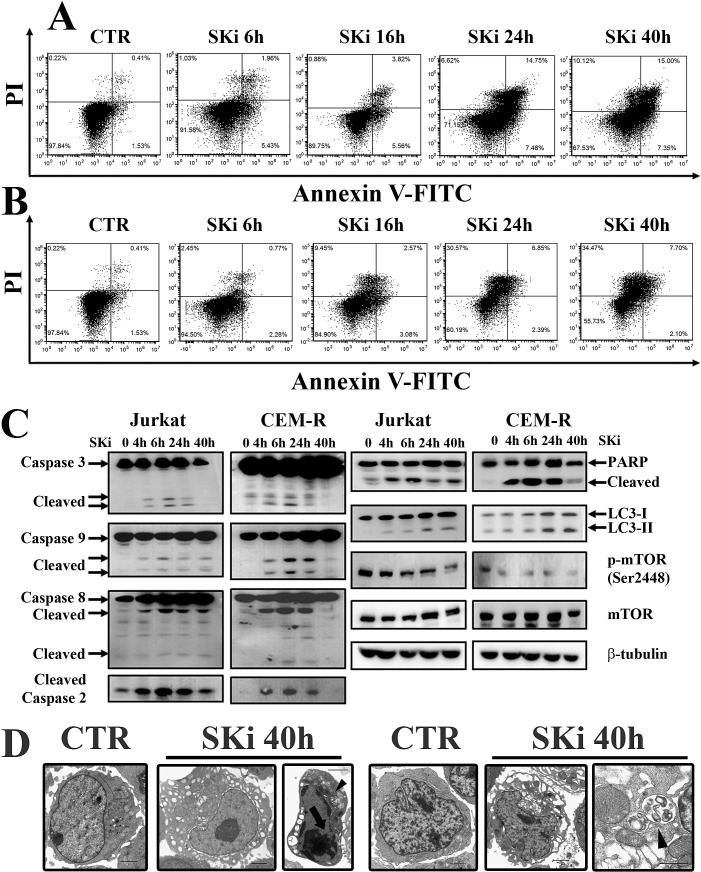
SKi treatment induces apoptosis and autophagy in Jurkat and CEM-R cells (A-B) Flow cytometric analysis of Annexin-V-FITC/PI staining in Jurkat and CEM-R cells, respectively. Cells were treated with SKi for 6, 16, 24, 40 h at the relative IC_50_. (C) Western blot analysis for apoptotic markers (caspase-3, -9, -8, -2 and PARP), autophagy marker (LC3) and p-mTOR (Ser2448). Protein (50 μg) was loaded for each lane.β-tubulin was used as a loading control. (D) Jurkat and CEM-R cell lines were treated with SKi for 40 h, and then TEM analysis was performed. Apoptotic features such as chromatin condensation (see arrows) and autophagic features such as vacuoles and autophagosomes (indicated by the arrowhead) were visible in Jurkat cells. Scale bar 2 μm; detail of autophagic vacuoles scale bar 1 μm.

We next assessed the effects of SKi on the death of Jurkat and CEM-R cell lines. SKi treatment of these cells not only augmented the percentage of early and late apoptotic cells, but also induced an increase in the population of cells that were stained by Annexin-V^−^/PI^+^ (Figure 3A and Figure 3B). Consequently, we analyzed whether SKi activated apoptotic and autophagic pathways in these cells. SKi activated apoptosis in Jurkat and CEM-R cells as demonstrated by the cleavage of caspase-3, -9, -8, -2, and PARP. Interestingly, in Jurkat and CEM-R cells, caspase-3 and caspase-9 cleavage peaked at 6 h and was less evident or absent after longer exposure to SKi. Therefore, consistent with the effect of SKi on cell viability, these results supported the possibility that a sub-population of Jurkat and CEM-R cells were resistant to apoptotic death as evident in Figure 1A at concentrations > 5 μM SKi, where there is no further decline in cell viability.

Interestingly, there was a delayed conversion from LC3 I to the lipidated form LC3 II at 24 and 40 h after addition of SKi to the cells. These findings suggested the activation of autophagy at later time points. Of note, there was a concomitant down-regulation of p-mTOR (Ser2448) that might release the inhibition of the autophagic process that can be exerted by the mTORC1 complex (Figure 3C) [26]. Therefore, the autophagic response occurred after the peak of apoptotic response. This is consistent with an adaptive protective mechanism that could account for the resistance to SKi in a sub-population of the T-ALL cells.

We also investigated the effects of SKi (40 h treatment) on the cellular structure and organization in Jurkat and CEM-R cells, using TEM analysis. SKi caused an enlargement of the endoplasmic reticulum (ER) compared to controls, that is characteristic of ER stress, and the appearance of autophagosomes containing cell components (Figure 3D).

**Figure 4:**
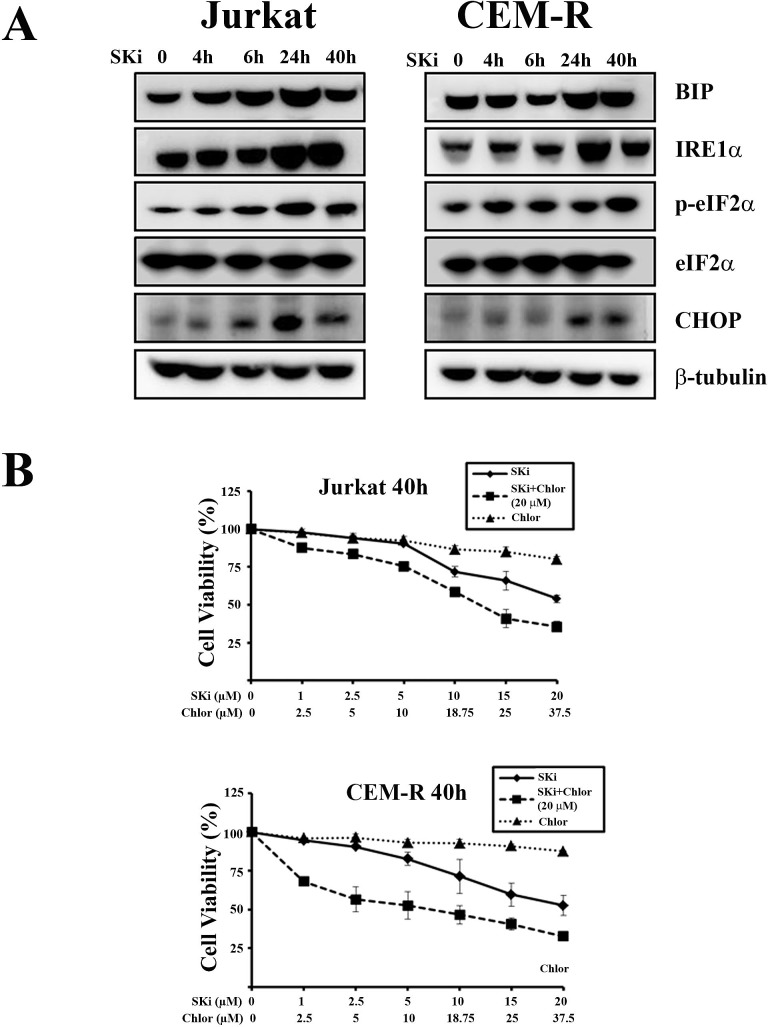
SKi induces autophagy as a consequence of ER stress/UPR activation in Jurkat and CEM-R cells (A) Western blot analysis for ER stress/UPR hallmarks in Jurkat and CEM-R cells treated with SKi for short (4 and 6 h) and long times (24 and 40 h). (B) MTT assays of T-ALL cell lines treated for 40 h with increasing concentration of chloroquine (Chlor), SKi, or increasing concentration of SKi with chloroquine (20 μM). Results are the mean of at least three different experiments ± s.d.

It is known that caspase-2 is implicated in the modulation of cell death induced by DNA damage, ER stress, and mitotic catastrophe [27]. It has also been documented that a relationship exists between ER stress and caspase-2 activation in human multiple myeloma cells [28]. In addition, it has been recently reported that ER stress and UPR activation drives apoptosis in T-ALL cell lines [29]. The cleavage of caspase-2 and an enlarged ER observed by TEM, prompted us to investigate if an UPR in Jurkat and CEM-R cells is initiated in response to SKi. We therefore analyzed the expression levels of several ER stress/UPR hallmarks, such as IRE1α, GRP78 (BIP), p-eIF2α and CHOP. SKi induced a substantial increase in the expression of these UPR proteins between 24 and 40 h, which confirmed the induction of ER stress/UPR. The time course of ER stress was post-apoptotic and correlated with autophagy (Figure 4A). Interestingly, CEM-S cells, which were less sensitive to SKi, displayed a stronger induction of IRE1α, and CHOP than CEM-R cells (Supplementary Figure 1). In contrast, Molt-4 cells, did not display any significant induction of ER stress/UPR hallmarks (Supplementary Figure 2).

Several lines of evidence have recently suggested a pro-survival effect of ER stress/UPR signaling pathway and autophagy in cancer [30, 31] while other studies support a tumor-suppressive role of UPR [32, 33]. In order to establish whether autophagy was a protective mechanism involved in maintaining ER homeostasis that subsequently promotes cancer cells survival, we treated Jurkat and CEM-R cell lines with chloroquine, an inhibitor of autophagy [34]. Treatment with increasing concentrations of chloroquine had little effect on cell viability after 40 h of incubation, whereas the combination of chloroquine (at the fixed concentration of 20 μM) and increasing concentrations of SKi induced a significant decrease in cell viability (Figure 4B). These findings indicated that the UPR/autophagic response is likely to be a protective response that might account for the escape of a sub-population of cells from SKi-induced death.

### SKi modulates signaling pathways involved in survival, proliferation and stress response

**Figure 5:**
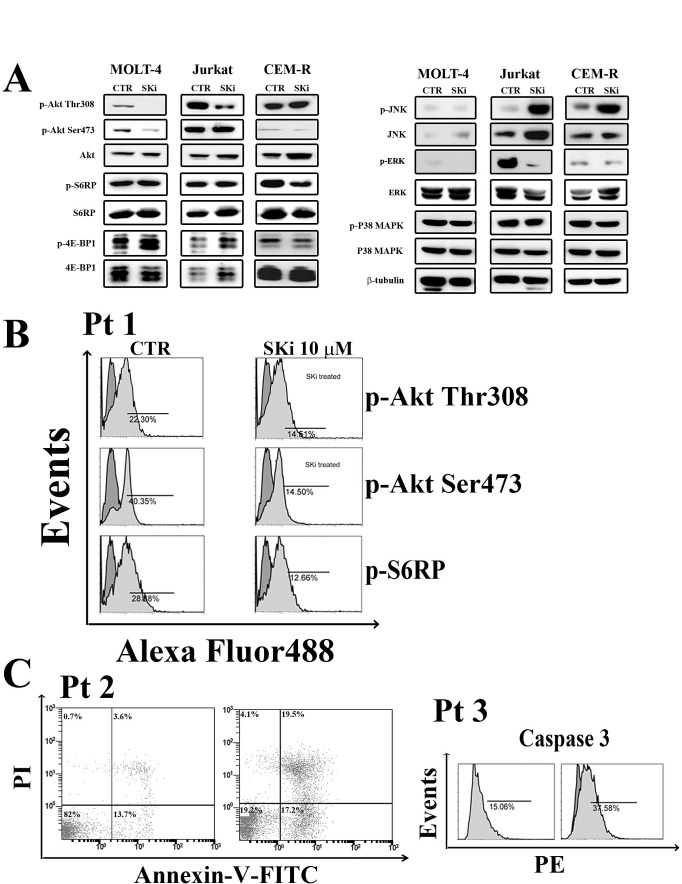
Effect of SKi on the phosphorylation status of signaling pathways controlling survival, proliferation and stress response. (A) Molt-4, Jurkat and CEM-R cells were treated with SKi for 40 h. Then, cells were collected, lysed, and analyzed by western blotting. Protein (50 μg) was loaded in each lane.β-tubulin was used as a loading control. (B-C) Primary cells from T-ALL patients were treated with SKi (10 μM) for 24 h, stained with various antibodies or Annexin-V/PI and subjected to cytometric analysis.

Many studies have shown that drugs targeting the S1P pathway can modulate crucial pro-survival signaling cascades such as PI3K/Akt/mTOR and Ras/Raf/MEK/ERK [23, 35, 36]. In addition, S1P is linked to cancer cell responses to stress such as stress-activated protein kinase/Jun-amino-terminal kinase (SAPK/JNK) and MAPK p38 networks [37]. Thus, we investigated whether SKi treatment affected these signaling pathways in T-ALL cell lines. Treatment of cells with SKi resulted in a decrease in the phosphorylation of Akt at both threonine 308 (Thr308) and serine 473 (Ser473) residues in Molt-4 cells. Indeed, ceramide, which might accumulate as a consequence of SK inhibition, has been shown to cause dephosphorylation of Akt, possibly via activation of PP2A [38]. However, SKi did not affect the phosphorylation of mTORC1 downstream targets S6 ribosomal protein (S6RP) and eukaryotic translation initiation factor (eIF)-4E-binding protein 1 (4EBP1) (Figure 5A). SKi treatment reduced the phosphorylation of Akt only at the Thr308 in Jurkat cells, while CEM-R cells were resistant to modulation of Akt phosphorylation by SKi. Interestingly, the phosphorylation of JNK was markedly increased by SKi in Jurkat and CEM-R cells and indicated that these cells are under stress. p38 MAPK signaling was not affected in all the three cell lines. However, the Ras/Raf/MEK/ERK cascade in Jurkat cell was profoundly reduced by treatment with SKi (Figure 5A), in agreement with others [23, 35].

Therefore, these findings demonstrated that SKi perturbed different signaling pathways which play crucial role in leukemogenesis [39], and this might provide a mechanistic explanation for the ability of SKi to induce apoptosis.

To establish whether our findings are relevant to human T-ALL, we performed experiments on lymphoblasts from T-ALL patients. Cells were treated with 10 μM SKi for 24 h and the status of the PI3K/Akt/mTOR signaling pathway was assessed. In agreement with the findings in T-ALL cell lines, SKi induced a decrease in the phosphorylation of Akt on both Thr308 and Ser473, as well as a decrease of p-S6RP levels (Figure 5B). SKi also induced apoptosis in T-ALL lymphoblasts as evidenced by Annexin-V/PI staining and flow cytometric analysis of cleaved caspase-3 (Figure 5C).

### SKi synergizes with chemotherapeutic drugs

**Figure 6:**
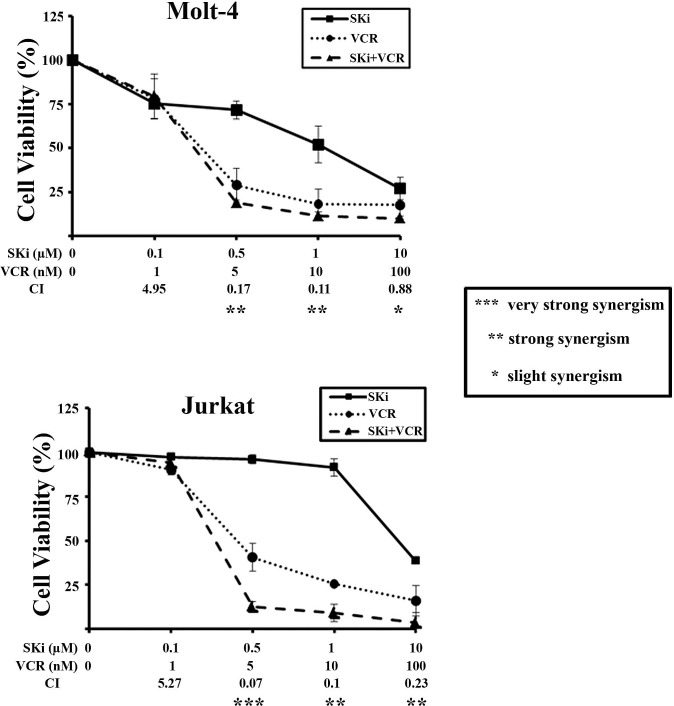
SKi and vincristine synergize in Molt-4 and Jurkat cells MTT assays of Molt-4 and Jurkat cells treated for 40 h with increasing concentrations of SKi and/or vincristine (VCR). The combined treatment resulted in strong synergism (CI < 0.3). Data represent the mean of at least three independent experiments ± s.d.

The ability of a sub-population of T-ALL cells to escape apoptosis in response to SKi prompted us to evaluate whether treatment of cells with SKi could potentiate the effect of established anti-cancer agents or *vice versa* to circumvent this problem. We used doxorubicin and vincristine (VCR), two drugs currently in use for treating T-ALL patients [40]. Molt-4, Jurkat and CEM-R cells were incubated for 40 h with increasing concentrations of SKi alone (0.1-10 μM) or with SKi (0.1-10 μM) in combination with increasing concentrations of VCR (1.0-100 nM). There was no observed synergistic effect between SKi and VCR in CEM-R cells as well as between SKi and doxorubicin at the concentrations we used in the three cell lines (data not shown). However, a strong synergism between SKi and vincristine was detected in Molt-4 and Jurkat cells. This occurred at concentrations of vincristine ranging from 5 to 10 nM in both cell lines (Figure 6A). Of note, the combination index (CI) analysis revealed that synergism occurred at concentrations of SKi that were significantly lower than its respective IC_50_ (synergism at 0.5 and 1 μM of SKi in Molt-4 and Jurkat cells), suggesting that vincristine sensitized T-ALL cells to SKi.

### ROMe causes autophagic cell death in T-ALL cell lines

**Figure 7:**
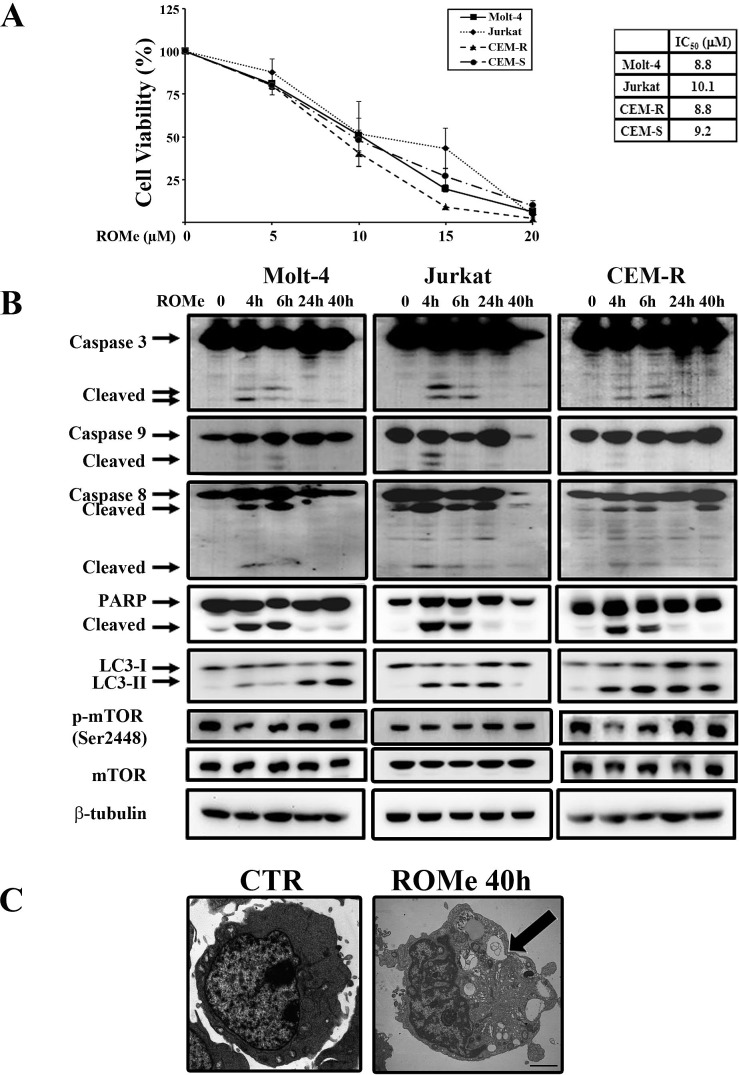
ROMe induces autophagy in Molt-4, Jurkat and CEM-R cells (A) MTT assays of Molt-4, Jurkat, CEM-R, and CEM-S cells treated with increasing concentrations of ROMe for 40 h. The results are the mean of three different experiments ± s.d. The table shows IC_50_ values of each cell line. (B) Western blot analysis documented that incubation with ROMe for 4, 6, 24, and 40 h activated caspases only after very short times of drug incubation, and a sustained autophagy in Molt-4, Jurkat and CEM-R cell lines. Cells were treated with ROMe at a concentration which corresponds to the IC_50._ Protein (50 μg) was loaded for each lane.β-tubulin was used as a loading control. (C) TEM analysis of CEM-R cells documented the presence of large cytoplasmic vacuoles containing various degraded organelles (arrow). Scale bar 2 μm.

Despite the controversial role of SK2 in apoptosis and cell fate, there is mounting evidence that SK2 is implicated in cancer. Indeed, several groups have described the anti-cancer activity of different SK2-selective inhibitors and SK2 siRNA in many types of tumors [13, 14, 20, 41]. Hence, we examined the effect of the SK2 inhibitor ROMe on the viability of T-ALL cell lines. We incubated cells with increasing concentrations of ROMe for 40 h. ROMe induced a reduction in cell viability that was concentration-dependent and with IC_50_ values of 8.8 μM for Molt-4 and CEM-R, 9.2 μM for CEM-S, and 10.1 μM for Jurkat cells (Figure 7A). Moreover, ROMe induced a complete reduction in cell viability suggesting that the cells are unable to mount a resistance response to this SK2 inhibitor.

**Figure 8:**
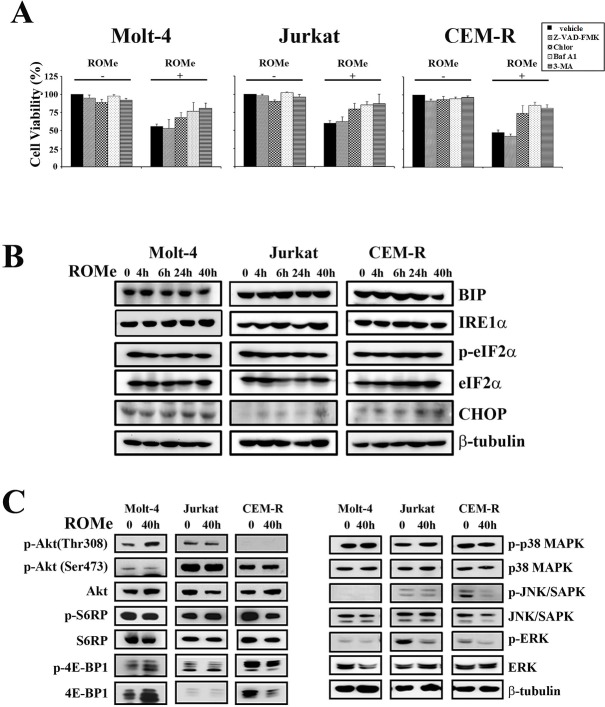
ROMe induces an autophagic cell death without the activation of the ER stress/UPR pathway (A) Inhibition of autophagy by chloroquine (Chlor) (20 μM), bafilomycin A1 (baf A1) (5 nM), or 3-MA (200 μM) [60] impairs the cytotoxic effect of ROMe (40 h). Inhibition of apoptosis through Z-VAD-FMK (20 μM) does not affect ROMe-induced cell death. Cell viability was assessed by MTT assay on Molt-4, Jurkat, and CEM-R cells. (B) Cells were treated with ROMe for 4, 6, 24 and 40 h, collected, lysed and subjected to western blotting for ER stress/UPR markers. Protein (50 μg) was loaded for each lane.β-tubulin was used as a loading control. (C) Molt-4, Jurkat and CEM-R were treated with ROMe for 40 h and western blot analysis was performed. ROMe does not interfere with the analyzed signaling pathways, with the exception of a down-regulation of Raf/MEK/ERK in Jurkat cells.

To understand the mechanism of action of the drug, we treated Molt-4, Jurkat and CEM-R cells with a ROMe concentration equivalent to the IC_50_ for 4, 6, 24 and 40 h and then analyzed the effect on apoptotic and autophagic pathways. Interestingly, the autophagic process was activated in a sustained manner, as evidenced by the accumulation of lipidated LC3-II (Figure 7B). Treatment of cells with ROMe also induced cleavage of caspase-3, -8 and -9, which peaked at 4 and 6 h, suggesting that apoptosis is activated only at very early time-points and the major cellular death is triggered by other mechanisms. Consequently, TEM analysis was performed on the three cell lines which revealed the typical autophagic features in Molt-4, Jurkat and CEM-R cells (Figure 7C). In addition, we demonstrated that, in all the three cell lines, exposure to the autophagic inhibitors chloroquine, 3-methyladenine (3-MA) and bafilomycin A1 markedly reduced the effect of ROMe on cell viability (Figure 8A), whereas treatment with the pan-caspase inhibitor Z-VAD-FMK does not affect it (Figure 8A), suggesting that autophagy is the major mechanism of cell death.

We next analyzed ER stress/UPR hallmarks. We observed that these markers associated with autophagic survival, did not exhibit any significant modulation, suggesting that the UPR response is specific to SKi, and possibly SK1 inhibition (Figure 8B) and that autophagy in response to ROMe represents a cellular death mechanism. Finally, we investigated whether ROMe modulates signaling networks that regulate cell survival. In contrast with SKi, ROMe did not affect any of the signaling pathways analyzed with the exception of a reduction in the levels of phosphorylated ERK in Jurkat and CEM-R cells (Figure 8C).

## DISCUSSION

A growing body of evidence indicates that sphingolipids play a critical role in regulating several physiological and pathophysiological processes, including cancer. Indeed, S1P is found in higher levels in many tumors compared with normal tissue and SK1 overexpression is associated with a poor prognosis in solid and hematological malignancies [1, 42]. Many efforts are being devoted to the development of new molecules targeting sphingolipid-based pathways. In recent years, many compounds have been synthesized and tested in *in vitro* and *in vivo* models of different type of cancers.

Among the chemical inhibitors that are selective for SK2 over SK1, the best are ABC294640 [13], ROMe [20], K145 [41], SLR080811 [43] and F02 [44], which have moderate potency, with Ki values in the range of ~1-10 μM. Moreover, treatment of MCF-7 breast cancer cells with ROMe prevented actin enrichment into lamellipodia in response to S1P, suggesting that migratory and metastatic responses of cancers cells might be dependent on SK2 [20]. In addition, ABC294640 suppressed the proliferation of several tumor cell lines, and inhibited tumor cell migration associated with the loss of actin microfilaments [13]. ABC294640 also displayed significant *in vivo* activity and inhibited tumor progression in mice with mammary adenocarcinoma xenografts [13]. More recent studies have demonstrated that ABC294640 induced autophagic death in A-498 kidney, PC-3 prostate and MDA-MB-231 breast cancer cells [36].

Despite excellent progress in the treatment of T-ALL, with most promising results obtained in pediatric patients, an efficient cure is still challenging. Mortality from T-ALL is still 25% for children and 40-50% for adults [16]. For this reason, innovative approaches that are more effective and display less toxicity, especially for patients who develop resistance to traditional chemotherapeutic drugs, are needed.

In this study, we investigated the therapeutic potential of two SK inhibitors in pre-clinical settings of T-ALL. In particular, we tested SKi and ROMe, that target SK1/SK2 and SK2, respectively, in T-ALL cell lines and primary cells. We demonstrated that both inhibitors induce a reduction in the cell viability with IC_50_ values that were comparable, but they exerted their effects through different mechanisms.

SKi initiated an apoptotic cell death in Molt-4, Jurkat, and CEM-R cell lines, as well as in lymphoblasts from T-ALL patients, in agreement with other studies [3]. Although an apoptotic process was initiated upon SKi treatment, a sub-population of T-ALL cell lines seems to escape death using different strategies. A ‘non-oncogene addiction' has been proposed for SK1, as it has been found over-expressed in many types of solid tumors [42]. In the present study, we reported that SKi induced increased expression of SK1 in Molt-4 cells. Since we know from cytometric analysis that, after 40 h, a large amount of cells die, it is possible that the remaining viable cells represented a sub-population that are more resistant to apoptosis owing to the adaptive increase in SK1 expression in these cells. This hypothesis is supported by the negligible induction of ER stress/UPR pathway in this cell line.

Jurkat and CEM-R cells behaved differently. We did not observe any increased SK1 expression in these cell lines. However, after 24 h of SKi treatment an ER stress/UPR pathway was initiated. UPR is a highly conserved stress pathway which is considered to be an adaptive response for safeguarding cell survival and that is aimed at restoring ER homeostasis [45]. It is known that the ER stress/UPR pathway is a potent stimulus of autophagy which represents an attempt by the cells to counteract the ER stress [30, 31].We report here that SKi induced the activation of an ER stress/UPR-induced autophagy in Jurkat and CEM-R cell lines. TEM analysis demonstrated the occurrence of alterations in ER structural organization and double membrane autophagosomes in the cytoplasm. These findings are consistent with an ongoing autophagic process. Consistently, LC3 conversion, another *bona fide* autophagic marker, was evident after 24 h of the drug treatment. Of interest, in Jurkat and CEM-R, but not in Molt-4 cell lines, we found a reduction of the phosphorylation status of mTOR (at Ser 2448) which is a negative regulator of autophagy and whose decreased expression might be associated with autophagy. Accordingly, treatment of Jurkat and CEM-R cells with chloroquine, an inhibitor of autophagy, potentiated rather than prevented cell death, suggesting a cyto-protective role of autophagy.

We also demonstrated that a combination of SKi with vincristine, a drug commonly used in T-ALL therapy, was synergistic in promoting T-ALL cell death. Therefore, SKi could potentiate the effect of chemotherapeutic drugs by surmounting the protective mechanisms, such as autophagy that confer resistance to SKi.

Interestingly, we reported that SKi treatment perturbed signaling cascades implicated in survival, proliferation and stress response of cells in our *in vitro* models. In particular, SKi treatment caused a reduction in p-Akt in Molt-4 and Jurkat cells. SKi could also modulate the Ras/Raf/MEK/ERK pathway, which is constitutively activated in Jurkat cells. This caused a marked decrease in p-ERK (Thr202/Tyr204) levels. In addition, there is a strong increase in p-JNK (Thr183/Tyr185) levels in Jurkat and CEM-R cells, suggesting that these cells are under a severe stress response. In fact, it is known that an increase in SAPK/JNK signaling pathway is a common response to many forms of stress, including ER stress (IRE1α-induced) [30].

We also assessed the effect of ROMe, a selective SK2 inhibitor, on T-ALL cell line survival. Recently, it was shown that the inhibition of SK2 activated autophagy in solid tumors [11, 13]. Despite an initial cleavage of caspases upon treatment of cells with ROMe, we found that ROMe induced a cytotoxic response through the activation of an autophagic process, as demonstrated by TEM analysis and increased LC3 processing. This hypothesis was then confirmed by treatment with the autophagic inhibitors chloroquine, bafilomycin A1 and 3-MA that were able to block the effect of ROMe, whereas the pan-caspase inhibitor Z-VAD-FMK was not. The concomitant cleavage of caspases, detected only after 4 and 6 h of ROMe exposure, is not surprising, given the strict interconnection between apoptosis and autophagy, as reviewed by many authors [46-48].

Notably, ROMe did not activate the ER stress/UPR pathway and did not perturb several signaling pathways including PI3K/Akt/mTOR, SAPK/JNK, and p38 MAPK. These data demonstrated that ROMe drives cell death through different mechanisms compared with SKi. More importantly, our findings demonstrated different cellular outcomes of autophagy that might be determined by the presence or absence of UPR and mTOR regulation.

In addition, the ability of ROMe to completely reduce T-ALL cell viability suggested that, in our *in vitr*o models, no adaptive protective mechanisms are activated by ROMe. This might suggest a more compelling therapeutic utility for SK2 selective inhibition over and above combined SK1 and SK2 inhibition in T-ALL cells.

In conclusion, the data presented here, strongly suggest that SK1 or SK2 inhibition is linked with a complex network of cell survival and death responses in T-ALL cells. These findings also provide useful information that could be used for designing novel SK2 inhibitors aimed at eradicating T-ALL cells and thereby enabling a better management of this type of cancer.

## MATERIALS AND METHODS

### Chemicals

Shingosine kinase (SKi) (2-(p-hydroxyanilino)-4-(p-chlorophenyl)thiazole) was purchased by Calbiochem (EMD Chemicals, San Diego, CA, USA). Vincristine and doxorubicin were from Sigma-Aldrich (St. Louis, MO, USA). Bafilomycin A1, 3-methyladenine (3-MA), and Z-VAD-FMK were from Enzo Life Science (Plymouth Meeting, PA, USA). ROMe ((*R*)-FTY720 methyl ether) was synthesized according to Lim and colleagues [20]. Antibody to SK1 was as reported elsewhere [7]. For western blot analysis, primary antibodies were from Cell Signaling Technology (Danvers, MA, USA). For flow cytometric analysis, phycoerythrin (PE)-conjugated anti-cleaved caspase-3 (Asp175) was from Cell Signaling Technology. p-Akt and p-S6RP levels were performed as reported elsewhere [49, 50].

### Cell culture and primary samples

The T-ALL cell lines Molt-4, Jurkat, CEM-R (CEM VBL100, drug-resistant cells overexpressing 170-kDa P-glycoprotein [51], and CEM-S were grown in RPMI 1640 supplemented with 10% heat-inactivated fetal bovine serum (FBS). Samples from T-ALL pediatric patients were obtained with informed consent according to Institutional guidelines and isolated using Ficoll-Paque (Amersham Biosciences, Little Chalfont, UK) and were grown in complete medium (RPMI 1640 supplemented with 20% FBS, and ITS (insulin-transferrin-sodium selenite).

### MTT assay

Cell viability was assessed using MTT (3-[4,5-Dimethylthythiazol-2-yl]-2,5- Diphenyltetrazolium Bromide) assays as previously described [52, 53]. In particular, T-ALL patient lymphoblasts (1 × 10^6^ cells/ml) were cultured in triplicate in flat-bottomed 96-well plates at 37°C with 5% CO2. Cultures were carried out for 48 and 96 h. Results were statistically analyzed by GraphPadPrism Software (GraphPad Software Inc., San Diego, CA, USA).

### Annexin-V/PI staining

Analysis of cell viability and apoptosis was carried out after drug treatment by dual staining with Annexin V-FITC and propidium iodide (PI) as reported elsewhere [54]. Cells were analyzed on a FC500 flow cytometer (Beckman Coulter, Miami, FL, USA).

### Western Blot analysis

Western blot analysis was carried out by standard methods, as previously reported [55, 56]. Analysis with an antibody to β-actin or β-tubulin demonstrated equal protein loading. For SK1 analysis, whole cell extracts were prepared as described [11].

### TEM analysis

TEM analysis was performed according to standard techniques, as previously described [57], using a Philips CM10 (Philips, Eindhoven, The Netherlands) TEM. Images were recorded on a Megaview III digital camera (Olympus, Tokyo, Japan).

### Combined drug effect analysis

The combination effect and a potential synergy were evaluated from quantitative analysis of dose-effect relationships as described previously [58, 59]. For each combination experiment, a combination index (CI) number was calculated using the Biosoft CalcuSyn software. This method of analysis generally defines CI values of 0.9 to 1.1 as additive, 0.3 to 0.9 as synergistic, and <0.3 as strongly synergistic, whereas values >1.1 are considered antagonistic.

## SUPPLEMENTARY MATERIAL FIGURES


