# Supplementary material for: Assessment of the effect of sphingosine kinase inhibitors on apoptosis,unfolded protein response and autophagy of T-cell acute lymphoblastic leukemia cells; indications for novel therapeutics
Source: Oncotarget. 2014 Aug 6;5(17):7886–901. doi: 10.18632/oncotarget.2318 (PMC4202168; doi:10.18632/oncotarget.2318)
Supplement: Supplementary file 1 [file oncotarget-05-7886-s001.pdf]

Assessment of the effect of sphingosine kinase inhibitors on apoptosis,unfolded protein response and autophagy of T-cell acute lymphoblastic leukenia cells; indications for novel therapeutics

Supplementary Material

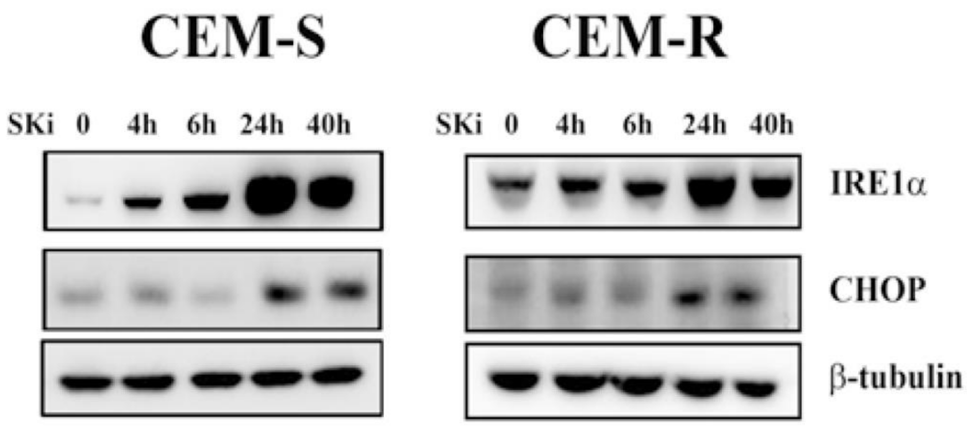

Supplementary Figure 1: Western blot analysis for IRE1α and CHOP in CEM-S and CEM-R cells treated with SKi for short (4 and 6 h) and long times (24 and 40 h). Fifty µg of protein was loaded for each lane. β-tubulin was used as a loading control.

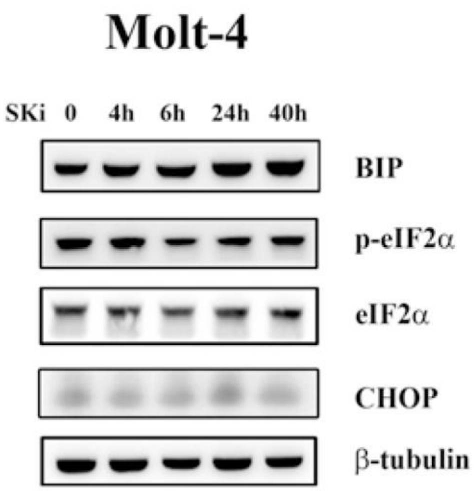

Supplementary Figure 2: Western blot analysis for BIP, p-eIF2α, total eIF2α, and CHOP in Molt-4 cells treated with SKi for 4, 6, 24, and 40 h. Fifty µg of protein was loaded for each lane. β- tubulin was used as a loading control.
